# Can Bisphenols Alter the Inflammation Process?

**DOI:** 10.3390/life15050782

**Published:** 2025-05-14

**Authors:** Denis Bazany, Hana Greifova, Lucia Zuscikova, Katarina Tokarova, Tomas Jambor, Anton Kovacik, Norbert Lukac

**Affiliations:** 1 Institute of Applied Biology, Faculty of Biotechnology and Food Sciences, Slovak University of Agriculture in Nitra, Tr. A. Hlinku 2, 949 76 Nitra, Slovakia; xbazany@uniag.sk (D.B.); xzuscikoval@uniag.sk (L.Z.); katarina.tokarova@uniag.sk (K.T.); tomas.jambor@uniag.sk (T.J.); norbert.lukac@uniag.sk (N.L.); 2 AgroBioTech Research Centre, Slovak University of Agriculture in Nitra, Tr. A. Hlinku 2, 949 76 Nitra, Slovakia; hana.greifova@uniag.sk

**Keywords:** inflammation, bisphenols, endocrine disrupting chemicals, prostaglandins, interleukins

## Abstract

This review’s main purpose is to draw attention to the possible influence of widely used bisphenols on the inflammatory process. Bisphenols are endocrine-disrupting chemicals that are produced worldwide in great quantities. From this point of view, it is very important to clarify their influence on innate immune reactions, which protect the integrity of the body against the action of various pathogens on a daily basis. The inflammation process consists of several key factors that are produced at different levels of this reaction. Each of these levels can be affected by endocrine disruptors, from the point of view of modifying either the immune system cells that intervene in this process or the way in which they produce inflammatory mediators. The development of new recommendations for the use of bisphenols is a complex issue given their influence on inflammatory processes. Because the immune system and immune response are so intricate, bisphenols may pose more risk to humans than is presently recognized. This paper discusses the classification of bisphenols, the fundamental mechanism of inflammation, the characterization of inflammatory mediators, and the current knowledge of the molecular mechanisms behind the impact of bisphenols on the inflammatory response.

## 1. Introduction

The immune system and inflammatory processes have been linked to a variety of physical and mental health issues that impact mortality and morbidity rates globally, which is one of the key medical discoveries of the last few years [1]. Inflammation represents an established phenomenon that is recognized as a key factor in infectious and chronic diseases [2]. Higher organisms have the ability to defend themselves against damaging stimuli such as microbial infections, damaged tissue, and other unpleasant situations by inducing an inflammatory response [3]. Throughout the inflammatory cascade, important microcirculatory processes include changes in vascular permeability, recruitment of leukocytes, their accumulation, and the release of inflammatory mediators [4].

Based on the duration of how the body responds to the injury, inflammation can be divided into three types: acute inflammation, which appears right after an injury and usually lasts a few days; chronic inflammation, which can linger for months or even years if acute inflammation fails to resolve; and subacute inflammation, which is a phase that occurs between acute and chronic inflammation and lasts for two to six weeks [5]. There are several types of inflammatory processes that are different in their triggering factor. Important triggers include, for example, lack of blood, immune system disorder, cancer, pathogen infection, physical injury, neurological factors, and last but not least, chemical exposure, which represents the main factor in the presented work [6].

This study mainly focuses on how a broad class of endocrine-disrupting chemicals known as bisphenols could possibly affect inflammation. Endocrine-disrupting chemicals (EDCs) are widespread compounds that are present in food, consumer goods, and the human environment. Scientists devote a great deal of attention to EDCs due to their potential hazards for human health [7,8,9].

**Figure 1:**
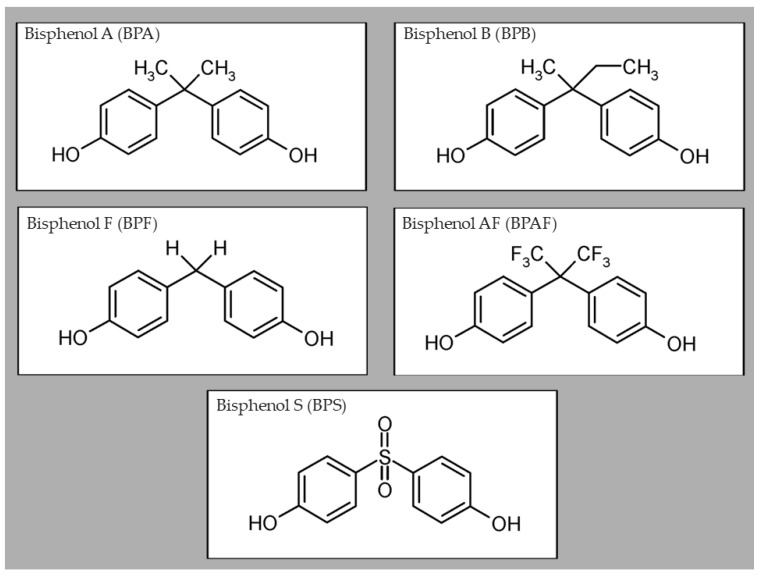
Comparison of the chemical structure of bisphenols, adapted from Frankowski et al., Applied Biochemistry and Biotechnology; Springer Nature (2020) [21].

Chemical substances known as bisphenols have one of the largest manufacturing volumes worldwide [10]. Many substances that structurally match bisphenol A are already utilized for the production of polycarbonate plastics and epoxy resins. Bisphenol A analogs, or BPA analogs, are comparable substances that have recently been adopted as alternatives to BPA. Seventeen bisphenols with the generic bisphenol structure are included in this category, as are bisphenol derivatives, which contain components with structural characteristics shared by bisphenols. Bisphenol A analogs are bisphenol AF (BPAF), bisphenol AP (BPAP), bisphenol B (BPB), bisphenol BP (BPBP), bisphenol C (BPC), bisphenol E (BPE), bisphenol F (BPF), bisphenol FL (BPFL), bisphenol G (BPG), bisphenol M (BPM), bisphenol P (BPP), bisphenol PH (BPPH), bisphenol S (BPS), bisphenol TMC (BPTMC), and bisphenol Z (BPZ) [11]. In this review, we discuss five of them (BPA, BPS, BPF, BPAF, and BPB; as shown in Figure 1), because these bisphenols are the most discussed in the scientific sphere and are also the most common industrial substituents.

Numerous bisphenol analogs display dioxin-like effects, neurotoxicity, cytotoxicity, reproductive toxicity, and effects that interfere with endocrine mechanisms in laboratory studies [12,13,14]. The estrogenic and/or anti-androgenic properties of bisphenol A (BPA) are matched or exceeded by those of its main substituents [15,16]. Because of these facts, bisphenol A and its analogs are not used as much as they could be because of immune, neurologic, reproductive, and other adverse effects associated with BPA. Replacement analogs are a present industrial option in large part due to lack of toxicity data so far generated for those analogs. BPA has been regulated, perhaps in the most noteworthy sense in Europe and in products to which human infants and children are exposed through dietary exposure. Due to the complexity of the immune system, it is necessary to focus not only on the well-described toxicity in terms of reproduction and the endocrine system but also on the potential immunotoxicity of bisphenols, which is still not sufficiently clarified and represents a possible health risk. The constant exposure to these substances, particularly from before birth and in the specific context of inflammation linked to the emergence of a wide range of chronic diseases, makes the modifications of inflammatory processes by bisphenols a formidable challenge when it comes to establishing new guidelines for their use.

## 2. Bisphenols

Bisphenols (BPs) are members of the diphenylmethanes family of chemicals, which are made up of two benzene rings divided by a central carbon atom and usually have a 4-OH substituent on both rings [17]. These endocrine-disrupting chemicals represent one of most common phenols found in the environment; all it takes to identify them is the presence of two phenols joined by an alkyl group. Although bisphenols are not naturally occurring in nature, they are now widespread because of their extensive production, consumption, and subsequent establishment in the environment [18]. These chemicals are produced by enclosed processes during the storage, handling, processing, and transportation of plastic products and are produced as a part of fugitive dust [19]. The bioaccumulation of BPs in the human organism, causing severe health implications, is a major concern since they migrate from contact between food materials and packaging materials including thermal sheets, epoxy sealants on metal cans, and plastic packaging [18]. For example, BPA is increasingly being used in kitchenware and utility goods due to its chemical characteristics, which include the great durability, outstanding heat stability, splinter resistance, and increased transparency of polycarbonate plastics. Additionally, it is used to make various food and drink packaging, reusable plastic bottles for newborns, medical equipment, small dishes, microwave-safe goods, tableware, dental sealants, and lacquers to cover metal objects like water pipes, bottle tops, and plastic cans, among other things [20].

### 2.1. Bisphenol A

Bisphenol A is a chemical substance with a crystalline structure that has been extensively used for more than 50 years as a key component in the production of epoxy resins and polycarbonate polymers [22]. It typically appears as a crystalline solid, colorless and white in ambient conditions, with a molecular weight of 228.29 g/mol with a light phenolic odor [10,23]. A compound’s molecular attributes (such as its molecular structure and weight) depend on its physicochemical qualities, which in turn determine the compound’s fate and dispersion in the environment [24]. It was anticipated that 10.6 million metric tons of BPA would be processed worldwide in 2022 [25]. Consequently, BPA has been found in drinking water at levels between 0.015 and 0.318 g/L and in water bottles at levels between 0.07 and 4.2 g/L [26].

BPA is persistently released and widely spread in the natural environment as a result of its massive production and extensive use [27]. BPA-based polycarbonate polymers have exceptional chemical and physical qualities, such as high resilience to acids and oils, toughness and durability, and thermal stability [28]. BPA is currently recognized as a hazard to human health because of its extensive dispersion, environmental durability, and reputation as a hormonal disruptor. Its potential links to cancer, metabolic disorders, heart difficulties, and infertility further raise concerns [29]. This compound could get into an organism through the skin, the lungs, or the digestive system, which is the most common way [30]. BPA is considered to have exhibited endocrine-disrupting effects via interacting through multiple biological receptors, including the thyroid hormone receptor (THR), androgen receptor (AR), and estrogen receptor (ER) [28].

The interaction of bisphenols with hormone receptors occurs through a mechanism of mimetic activity, where the bisphenol molecule binds to estrogen receptors (ERα, ERβ) and triggers estrogen-like signaling [31]. Bisphenol A has been the subject of public concern and regulatory legislation, which has sparked the production of substitute materials for BPA in a variety of applications [15]. Since plastic materials and products intended for food contact potentially release chemical compounds, the European Legislation specifies migration limitations for all permitted substances [32]. Concerns over extensive human exposure and associated health concerns have prompted European Union prohibitions on the fabrication and application of BPA [15]. The tolerable daily intake (TDI) for BPA has been set by the European Food Safety Authority at 0.02 ng/kg body weight/day, but some studies show effects even at lower concentrations [33]. The problem is aggregative exposure from multiple sources and synergistic effects with other chemicals (phthalates, pesticides) [34]. As of 2020, the amount of BPA in thermal paper is limited to 0.02% by weight [35]. These compounds pose ethical challenges. If a chemical does not have significant effects at low doses, regulators may allow it to be released into circulation or the environment, provided that procedures and rules are in place to keep exposure below acceptable levels. BPA exposure has been linked to hypertension and cardiovascular diseases in addition to endocrine disruption, oxidative stress, inflammation induction, and associations with other chronic illnesses [36].

### 2.2. Bisphenol S

A sulfonyl group is positioned between two phenol functional groups in bisphenol S (BPS), a compound whose chemical structure is comparable to that of bisphenol A [37]. At room temperature, it is usually colorless and crystalline with a molar mass of 250.27 g/mol. BPS is readily dissolved in acetone, ethanol, isopropyl alcohol, acetonitrile, and aliphatic hydrocarbons. In aromatic hydrocarbons, it dissolves weakly [38]. This endocrine-disrupting chemical has numerous industrial uses, such as a wash fastening agent in cleaning solutions, an electroplating solvent, and a component of phenolic resin [39]. In contrast to BPA, however, BPS has a longer half-life and is not amenable to biodegradation [40]. Still, BPS has been established as a main substitute to bisphenol A in industry applications. Research into the health impacts of BPS is still expanding, and mounting evidence indicates harmful effects that are comparable to those of BPA [41]. Because BPS has better physical qualities than BPA, it was assumed that it would be less prone to leaking monomers into food and beverages [42]. Additionally, a major source of BPS in consumer products is recycled paper and plastic, especially thermal paper [38]. However, since BPS has become more prevalent in society, as many as 81% of people living in the United States and Asia have tested positive for BPS exposure in their urine [43]. According to Regulation (EU) No. 10/2011, bisphenol S may be used as a monomer in plastic food contact materials, with a specific migration limit of 0.05 mg/kg food [44]. According to the European Chemicals Agency, the yearly production or import rate of BPS in the European Economic Area has reached 1000–10,000 tons [38].

### 2.3. Bisphenol F

Bisphenol F, also known as bis(4-hydroxyphenyl)methane is structurally composed of two phenol rings connected by methylene [45]. Broadly utilized in the production of consumer goods, bisphenol F (BPF) is an endocrine-disruptive chemical with a molecular weight of 200.23 g/mol. BPF is also used in numerous industrial applications, such as can lacquer coatings, food packaging, sealants, and dental composites. It is utilized in the production of epoxy resins and coatings, as well as polymers that impart increased thickness and durability to materials. In addition, epoxy resins are used in the production of numerous consumer goods in everyday use [41]. Like BPA, BPF is an endocrine disruptor with similar estrogenic, androgenic, and anti-estrogenic effects in a variety of in vitro tests. This chemical compound causes DNA damage by epigenetic changes and modifies signaling pathways related to adipogenesis and lipid metabolism differently [46]. BPF is able to penetrate the placental barrier and enter the fetus. It has been isolated from several kinds of organs, including the reproductive system [47]. Humans may be continuously exposed to low concentrations of BPF due to the free monomers of this bisphenol being released into the environment and entering the food chain [48]. BPF’s presence in the environment has significantly increased due to its broad application in numerous fields. A significant carrier of these harmful substances is water [49]. As BPF usage and environmental detection levels continue to rise, immediate consideration must be given to its environmental health effects [50]. According to the 2013–2014 National Health Nutrition Examination study, BPF was found in over 60% of urine samples in United States, with a geometric mean of 0.532 μg/L across all age groups [51]. In Chinese adults, its estimated daily food consumption was determined to be 102 ng/kg bw/day for females and 96.5 ng/kg bw/day for males [52].

### 2.4. Bisphenol AF

Bisphenol AF, also known as 1,1,1,3,3,3-hexafluoro-2,2-bis (4-hydroxyphenyl) or BPAF, structurally consists of a carbon bridge joining two phenolic rings [53]. A range of polymers used in optical fibers and electronic devices, as well as gaskets and food processing equipment, are made with the addition of BPAF, which is utilized in these applications as a monomer as well [54]. BPAF is one of the most prevalent substitutes for BPA, and it is extensively applied in the production of food packaging, electronic products, and optical fibers [55]. It has been found in both environmental and human samples, mainly urine, as well as in drinking water [56]. By interfering with neurological, immunological, reproductive, and endocrine function, accumulating BPAF causes toxicity in major organs such as the liver, brain, kidneys, and immune and reproductive systems [25]. Humans are exposed to BPAF through trace emissions from consumer products; detectable levels may be discovered in solid samples, food, drinks, indoor dust (739 ng/g), and ambient water (15.3 μg/L). Furthermore, BPAF has been found in human urine, serum and breast milk (>0.173 ng/mL, >0.404 ng/mL, and >0.58 ng/mL, respectively) [57,58].

### 2.5. Bisphenol B

Bisphenol B (BPB), (2,2-bis(4-hydroxyphenyl)butane) is structurally different from BPA in that, instead of a methyl group, an ethyl group is present on the central carbon atom. Compared to BPA, this compound’s aerobic and anaerobic biodegradation was shown to be slower [59]. BPB’s main physiological action is decreasing levels of cortisol and corticosterone; higher concentrations of BPB can cause DNA damage, and it also has considerably higher acute toxicity than BPA [60]. This endocrine-disrupting chemical has been proven to be a compound with estrogenic and anti-androgenic properties [61]. With growing quantities, BPB has become one of the most commonly identified bisphenols in the environment, food, and drinks [62]. Studies of human exposure to BPB are exceedingly limited, and BPB remains among the least examined and detectable bisphenols in the environment. Therefore, more research should be conducted to determine how BPB affects the human organism [63].

## 3. Elementary Process of Inflammation

The human body is continuously exposed to harmful stimuli from the surrounding environment. It has developed many ways to detect and respond to such stimuli and repair itself in order to preserve homeostasis throughout its evolutionary development. Inflammation is the adaptive reaction to any of these stressors, including infection, trauma, surgery, burns, ischaemia, and necrotic tissue [64]. On the basis of visual observation, our ancients identified five cardinal indications of inflammation: redness (rubor), swelling (tumor), heat (calor), pain (dolor), and loss of function (functio laesa) as result of chronic inflammation [65]. Inflammatory and immune responses are the primary mechanisms of protection against multiple externally damaging and endogenously abnormal genetic and biochemically imbalanced disease occurrences [66].

In the past, infections and the immune system were associated with inflammation. More recent evidence reveals that inflammatory indicators are present in a much wider range of diseases. The main process that allows tissue to recover after an injury has its basis in the inflammatory process. Tissue damage is eliminated and new tissue is produced by a series of cellular and microvascular mechanisms that involve inflammation [67]. The most prevalent polyunsaturated fatty acid in the human body, arachidonic acid, is the initial step in this cascade. Enzymes cyclooxygenase (COX), lipoxygenase (LOX), and cytochrome P450 (CYP450) are mainly responsible for the processing of arachidonic acid (AA), a significant component of cell membrane lipids. A variety of inflammation-stimulating metabolites might be produced from AA depending on these three metabolic pathways [68]. Many of the produced soluble mediators control how sedentary cells (endothelial cells, fibroblasts, and mast cells) and called-up inflammatory cells (like monocytes and neutrophils) are activated. Several of these mediators also ultimately cause cardinal reactions of the system to the inflammatory process (like fever, hypotension, leukocytosis, and output of acute phase proteins) [69].

In the scientific literature, there is evidence of the modulatory effect of bisphenols on the inflammatory process. Circulating levels of pro-inflammatory interleukin (IL)-6 and C-reactive protein (CRP) were positively correlated with BPA environmental exposure in human samples [70]. Also a number of other pro-inflammatory biomarkers, including IL-4, IL-17A, IL-23, IL-33, MCP-1, ALT, and γ-GTP, were positively correlated with BPA exposure [71]. Many studies suggest that bisphenols can modify the production of cytokines and thus alter the setting and course of the inflammatory cascade [72,73,74,75]. The findings show that BPS and BPF, which have comparable effect concentrations, function through different immunological pathways. BPS mainly targets macrophages to mediate immune responses, whereas BPF strongly promotes neutrophil proliferation and uses chemokine signaling to elicit a more potent inflammatory response [76]. Since bisphenols may activate the nuclear estrogen receptor (ER) α and generate inflammatory conditions, it has been claimed that inflammation might serve as a different or supplementary mode of action to the endocrine disruption principle. Accordingly, prior research has documented the presence of estrogen-dependent nuclear receptors in the promoter regions of genes linked to the inflammatory response, including ERα and ERβ. This suggests that the onset and progression of an inflammatory response may also be an indirect result of endocrine changes induced by these hormonally active substances [71].

### 3.1. Arachidonic Acid

Arachidonic acid (AA), which is also termed eicosa-5,8,11,14-tetraenoic acid, is a polyunsaturated fatty acid (PUFA) that is mostly found in the cell membrane as part of the phospholipids bilayer [68]. The body stores arachidonic acid in the lipid bodies of immune cells or as an integral part of the structural phospholipids in cell membranes [77]. This polyunsaturated fatty acid contributes to inflammation, causes blood vessels to dilate, and gives cell membranes their flexibility and fluidity. It also functions as a lipid second messenger in cell signaling [78].

This membrane compartment plays a role in maintaining the proper fluidity of mammalian cell membranes at normal temperatures. Arachidonic acid’s tendency to react with molecular oxygen is likewise based with the double bonds structure. There are multiple mechanisms for this to occur, including nonenzymatic mechanisms that promotes oxidative stress and the actions of cytochrome P450, lipoxygenase (LOX), and cyclooxygenase (COX). Arachidonic acid, even in its unaltered state, exhibits biological activity, as do the products of these enzymes and of the nonenzymatic conversions [78]. AA and its derivatives relate inflammation and immunity to the metabolism of nutrients. They are crucial to the onset and advancement of prevalent illnesses including diabetes, obesity, non-alcoholic fatty liver disease, and heart disease [79].

### 3.2. Phospholipase A2

Phospholipase A2 (PLA2) is the regulator of the eicosaniod metabolic pathway. It controls the release of polyunsaturated fatty acids, including arachidonic acid, from the sn-2 position of membrane phospholipids [80]. PLA2 has two types of isoforms, termed secretory and cytoplasmic. Because there are multiple isoforms, different tissues can respond in different ways. TNF-α, granulocyte-macrophage colony-stimulating factor, interferon (IFN), and several growth factors, including phosphokinase C and mitogen-activated protein kinase (MAPK), can all trigger PLA2 isoforms [81]. Cyclooxygenases and lipoxygenases, which are essential for the synthesis of eicosanoids such as leukotrienes, prostaglandins, and thromboxanes, are involved in a series of cellular processes that are induced by the release of AA and other PUFAs [82].

### 3.3. COX-1 and COX-2 Pathways

Exhibiting both cyclooxygenase and peroxidase activity, cyclooxygenase is a bifunctional enzyme. By adding two oxygen molecules to arachidonic acid, it cyclizes to produce cyclic prostaglandin G2 (PGG2), which the peroxidase process subsequently converts to hydroxyendoperoxide prostaglandin H2 (PGH2) [83]. The COX-1 and COX-2 isoenzymes accelerate the prostanoid synthesis process’s rate-limiting phases. The isoenzymes encoded by the COX-1 and -2 genes exhibit distinct activity but overlapping patterns of expression. Physiologically, the transcriptional and post-transcriptional expression of COX-1 and -2 genes are regulated by external stimuli such as growth hormones, cytokines, and tumor promoters [84]. The constitutively generated COX-1 subtype is normally considered a housekeeping enzyme, while the inducible COX-2 subtype has been associated mainly with the inflammatory process [85].

#### 3.3.1. COX-1

Cyclooxygenase-1 is a ubiquitously expressed enzyme with constitutive expression. The physiologic functions of prostaglandins and thromboxanes produced by the COX-1 pathway include platelet aggregation, gastric mucosal protection, and renal electrolyte metabolism [86]. It is widely understood that COX-1 activity keeps prostanoid production at an appropriate rate and allows for a rapid increase when cell membrane remodeling elevates the amount of free AA [87]. COX-1 has pharmacological significance as it is the target of acetylsalicylic acid (aspirin) and other nonselective nonsteroidal anti-inflammatory drugs (NSAIDs) [88].

#### 3.3.2. COX-2

COX-2 is an enzyme that may be stimulated by cytokines and growth hormones. Induction of COX-2 is associated with inflammatory cells and tissues and is thus present in the kidney and brain [89]. In contrast to COX-1,the cyclooxygenase-2 gene has several regulatory sites, making it possible for bacterial endotoxins such as lipopolysaccharide (LPS), cytokines such as interleukin (IL)-1 and tumor necrosis factor (TNF), growth factors (GFs), and the tumor promoter phorbol myristate acetate (PMA) to quickly induce COX-2 production [86]. To control the anti-inflammatory and anti-neoplastic characteristics of the COX-2 isoenzyme, a number of COX-2-selective inhibitors are currently being researched. Future anti-inflammatory and cancer therapies may be based on the suppression of COX isoenzyme expression and/or activity [84].

#### 3.3.3. Lipoxygenases

By generating pro-inflammatory mediators called leukotrienes or anti-inflammatory mediators called lipoxins, lipoxygenases—a class of oxidative enzymes with a non-heme iron atom in their active site—control inflammatory reactions. The addition of oxygen (O_2_) to polysaturated fatty acids (PUFAs), including linoleic acid and arachidonic acid, is catalyzed by these enzymes. The iron atom at the active site of lipoxygenases has been described as undergoing a single electron oxidation throughout the catalytic process, switching between the Fe^2+^ and Fe^3+^ redox states [90]. This enzyme, which is present in bone marrow-derived immune cells such neutrophils, monocytes, macrophages, dendritic cells, and mast cells, catalyzes the transformation of arachidonic acid into leukotriene A4. Leukotriene A4 is then either enzymatically hydrolyzed to generate leukotriene B4 or conjugated with glutathione to form leukotriene C4. Leukotriene C4 and its metabolites, leukotrienes D4 and E4, are representative examples of cysteine-containing leukotrienes [91].

## 4. Inflammation Mediators

Inflammation mediators are soluble, diffusible chemicals that have a targeted effect at the site of infection and tissue injury as well as in anatomically more distant locations. These mediators are also divided into groups by biochemical aspects:

1. Vasoactive amines (mainly histamine and serotonin)—primary local vasodilation and vasoconstriction
2. Lipid mediators (eicosanoids)—derived from arachidonic acid metabolism
3. Products of the complement system—inactive parts of the complement that can be activated by immunocomplexes
4. Cytokines involved in inflammatory responses
5. The kinin–kallikrein system and vasoactive peptides (bradykinin and lysyl-bradkinin)
6. Chemokines (chemotactic cytokines and chemotactic factors)
7. The hemocoagulation system and involved proteolytic enzymes

### 4.1. Vasoactive Amines

Several immune-system chemicals are capable of influencing blood flow. One of the most important of these are vasoactive amines. Due to their propensity to generate vascular permeability, vasodilation, and smooth muscle contraction, these substances are essential for the inflammatory response. Histamine and serotonin are the major vasoactive amines with this capability [92].

Mast cells are the primary source of histamine and display an abundance of surface receptors [93]. Histamine (2-[3H-imidazol-4-yl]ethanamine) is a crucial chemical mediator that promotes vasodilation, enhances vascular permeability, and may potentially play a role in anaphylactic responses. In addition, it impacts other physiological processes, including cell differentiation, proliferation, hematopoiesis, and cell regeneration [94].

Serotonin, also known as 5-hydroxytryptamine (5-HT), is a neurotransmitter and signaling molecule generated from tryptophan [95]. Serotonin is rapidly generated by mast cells, basophils, and platelets in response to injury. At the same time, activation of the complement system and the release of inflammatory substances like immunoglobulin E complexes and platelet activating factor (PAF) lead to chemotaxis, phagocytosis, and inevitably inflammation [96].

### 4.2. Lipid Mediators (Eicosanoids)

Eicosanoids are physiologically active lipids involved in several pathogenic mechanisms, including inflammation and cancer development [97]. The cytochrome P450 (cytP450), lipoxygenase (LOX), and cyclooxygenase (COX) pathways convert 20-carbon polyunsaturated fatty acids into eicosanoids. Typically, arachidonic acid serves as the substrate for eicosanoid production. Leukotrienes (LTs) and lipoxins (LXs) are produced by LOX metabolic pathways, prostaglandins (PGs) and thromboxanes (TXs) resulting from COX-involving processes, and other epoxy, hydroxy, and dihydroxy derivatives resulting from the action of cytP450 [98].

#### 4.2.1. Prostanoids (Prostaglandins and Thromboxanes)

**Figure 2:**
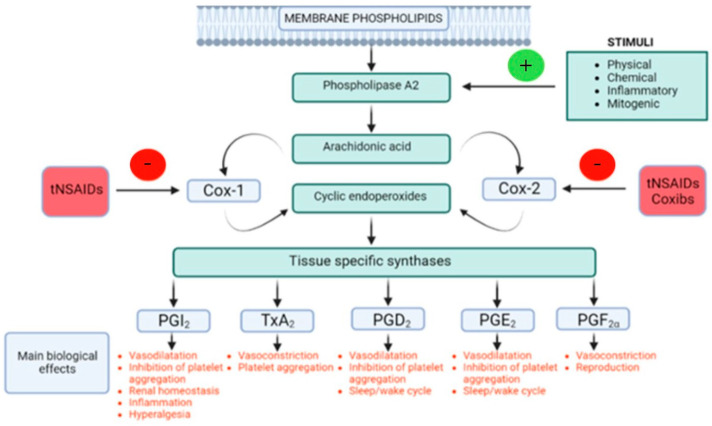
The primary physiological functions and biosynthesis of prostanoid mediators, as well as the site of NSAID activity, adapted from Brune et al., Journal of Pain Research; Taylor & Francis Group, (2015) [88].

Prostaglandins are structurally small lipid molecules that affect a variety of biological functions, such as proper kidney function, platelet aggregation, releasing of neurotransmitters, and immunoregulation [99]. They are frequently produced; typically, one or two primary products are generated by each kind of cell. They function as autocrine and paracrine lipid mediators to preserve local homeostasis in the body. The amount and character of PG production are significantly changed during an inflammatory response. In non-inflammatory tissues, PG production is frequently very low prior to the recruitment of leukocytes and the infiltration of immune cells, but it rapidly increases in acute inflammation [100]. The cyclooxygenase pathway utilizes arachidonic acid metabolism for the generation of prostaglandins. TXA2 (thromboxane), PGD2, PGE2, PGF2α, and PGI2 (prostacyclin) are the most significant forms of prostaglandins [101]. The biosynthesis and the main biological activities of prostanoid mediators, and the site of action of NSAIDs, are summarized in Figure 2.

##### Prostaglandin D2

PGD_2_ mediates several physiological effects in diverse organs and tissues, such as limiting platelet aggregation, vasodilation, and influencing the sleep/wake cycle. It is an important lipid mediator that promotes inflammation and is a byproduct of the cyclooxygenase/arachidonic acid pathway [102]. Numerous organs, including the heart, eye, brain, and epithelial cells, contain this prostanoid, which is also often produced in body fluids including plasma, cerebrospinal fluid, and eye secretions [103]. PGD synthases (PGDSs) convert PGH2 to PGD2, and COXs oxygenate arachidonic acid to PGH2 as part of a multi-enzymatic cascade event. Hematopoietic PGDS (H-PGDS) and lipocalin-type PGDS (L-PGDS) are in fact the two main categories of PGD2 synthases [104]. L-PGDS is expressed primarily in endothelial cells and cardiomyocytes of the cardiovascular system [105]. H-PGDS is extensively expressed in antigen-presenting cells such as mast cells and macrophages [106]. Two G-protein-coupled receptors, d-type prostanoid receptors 1 (DP1) and 2 (DP2), both of which are also known as the chemoattractant receptor homologous to Th2 cells (CRTH2), are stimulated by PGD2 release [107]. Leukocyte populations and the vascular system also include significant amounts of DP1, which when activated produces cytokines, vasodilation, bronchodilation, or platelet anti-aggregation [108]. By increasing intracellular Ca^2+^ mobilization and chemotaxis, CRTH2 promotes the activation of Th2 cells, eosinophils, and basophils [109]. According to a number of studies, PGD2 receptor-mediated signaling plays an essential role in asthma development and allergic lung inflammation [110].

##### Prostaglandin I2

Prostaglandin I2 (PGI), also known as prostacyclin, is a prostanoid well recognized for its activities in modulating inflammation. It does, however, play a variety of important roles in maintaining homeostasis and is thus a vasodilator and a powerful platelet aggregation inhibitor [111]. PGI is largely produced by vascular endothelium and smooth muscle cells, although it is also produced by fibroblasts, follicular dendritic cells, and thymic nurse cells [112]. During physiological processes, PGI2 is implicated in inflammatory reactions and CD4+ T cell activation [113]. Furthermore, it is an important immunoregulatory lipid mediator that influences the differentiation of Th17 and T-regulatory cells [114]. PGI synthase (PGIS) converts PGH2 into PGI2, and PGIS is predominantly, but not solely, found in endothelial cells, where it is most abundantly expressed [100].

##### Prostaglandin E2

The most common prostaglandin produced in both physiological and pathogenic conditions is PGE2, which has long been known to have immunosuppressive properties due to its ability to inhibit T cell activation [115]. The well-established inhibitory effect on Th1 differentiation and the suppression of macrophage production of inflammatory cytokines like IL-1 and TNF are partly responsible for this process [99]. PGE2 has been demonstrated to reduce both innate and Ag-specific immunity at multiple states of molecular and cellular levels, despite increasing the activation, maturation, and migration of dendritic cells (DCs), the key cells in the development of Ag-specific immunity [116]. All types of cells in the body can generate PGE2; however, during an immunological response, the main PGE2 producers are fibroblasts, epithelia, and infiltrating inflammatory cells [117]. Subsequently, the produced PGE2 will bind to many downstream prostaglandin E receptors located on the cell or organelle membrane, including EP1, EP2, EP3, and EP4, to exert its complex and varied physiologic effects [118]. EP receptors belong to a broad family of seven transmembrane domain receptors that activate different second messenger signaling pathways by binding to certain G proteins. The expression of each EP receptor and the intensity of each EP signal define the ultimate outcome of PGE2 signaling [119].

##### Prostaglandin F2α

PGF2 is an important signaling factor in parturition because it not only stimulates uterine contraction but also amplifies pro-inflammatory cytokine and chemokine production [120]. All enzymes that generate PGF2 belong to the aldo-keto reductase (AKR) family [121]. The activity of AKR1C3, also known as PGF synthase, may directly convert PGF2a from PGH2 [122]. PGF2’s actions are mediated through a seven-transmembrane G-protein-coupled receptor (FP) [123]. This prostaglandin can affect luteal cell viability by promoting proliferation or cell death through apoptosis or necrosis, depending on its local and systemic effects [124]. Numerous studies have revealed that pure exogenous PGF2 or a synthetic counterpart has a substantial chemoattractant effect [125].

##### Thromboxane A2

Thromboxane A2 (TxA2) was formerly thought to be secreted by platelets, but it is now recognized to be released by a wide range of different cells such as macrophages, neutrophils, and endothelial cells. Named for its function in thrombosis, TxA2 has pro-thrombotic characteristics because it increases platelet activation and aggregation and is an acknowledged vasoconstrictor that is induced during tissue damage and inflammation [126]. The TP receptor on the cell surface allows TXA2 to control biological processes. Its receptors are activated by TAX2 and isoprostanes. Thromboxane and its receptor levels are increased in a variety of cardiovascular and inflammatory disorders [127]. TXA2 is synthesized from PGH2 by the terminal enzyme TXA2 synthase (TXA2S) [128]. Although TXA2S is abundantly expressed in the lungs, kidneys, stomach, duodenum, colon, and spleen, it was initially discovered as a microsomal enzyme in platelets (60 kDa) [129].

#### 4.2.2. Leukotrienes

Leukotrienes (LTs) are physiologically active lipid mediators derived from arachidonic acid oxidative metabolism. The group of soluble and membrane-bound enzymes that make up an apparatus complex that is mostly produced by myeloid cells are involved in the production of leukotrienes. The immunological regulation of leukocyte migration is significantly influenced by leukotrienes and the synthetic enzymes that produce them. Numerous inflammatory conditions have been connected to elevated leukotriene levels [130]. LTs are classified as either cysteinyl leucotrienes (Cys-LTs; LTC4, LTD4, and LTE4), which additionally include amino acid moieties, or chemoattractant LTB4, which merely contains hydroxyl moieties [131]. They bind to G-protein-coupled receptors (GPCRs) and cause their biological effects. The roles and patterns of expression of LT receptor subtypes vary. BLT1 and BLT2 LT receptors are activated by leukotriene B4 (LTB4), while CysLT1 and CysLT2 receptors are activated by cysteinyl LTs (Cys-LTs) [132].

Leukotriene B4 (LTB4) is a potent lipid mediator that plays a crucial role in tissue damage and inflammation by recruiting and activating neutrophils [133]. LTB4 receptor 1 (BLT1), a high-affinity G-protein-coupled receptor for LTB4, is primarily responsible for mediating the potent biological effects of LTB4 [134]. BLT1 is expressed primarily in dendritic cells, monocytes, differentiated T cells, neutrophils, and macrophages and their progenitors [135].

The slow-reacting substance of anaphylaxis (SRS-A) has been shown to include the cysteinyl leukotrienes leukotriene C4, leukotriene D4, and leukotriene E4, which cause smooth muscle contraction and enhanced vascular permeability. In the pathophysiology of inflammatory illnesses, these compounds are potent biological mediators that cause contractile and inflammatory processes through specific interactions with cell surface receptors that are members of the G-protein-coupled receptor superfamily [136].

#### 4.2.3. Lipoxins

Endogenous anti-inflammatory chemicals called lipoxins have an essential role in reducing chronic inflammation and tissue damage [137]. The three main lipoxygenases (LOs) that convert arachidonic acid into lipoxins are 5-LO, 15-LO, and 12-LO. Platelets undergo the first step of lipoxin synthesis when 12-LO interacts with leukotriene A4 to transform it into lipoxins [138]. The initial process of lipoxin production occurs in platelets, where 12-LO acts on leukotriene A4 and converts it to lipoxins. A sequence of LO acts on arachidonic acid, including 5-LO in neutrophils and 15-LO in erythrocytes and reticulocytes, forming the second pathway of lipoxin synthesis. Following its conversion from arachidonic acid to 15-hydroxyeicosatetraenoic acid, lipoxin A and lipoxin B are merged [139]. The third process produces 15 epi-lipoxin B4 and 15 epi-lipoxin A4, sometimes referred to as aspirin-triggered lipoxin (ATL) [140].

### 4.3. Cytokines Involved in Inflammatory Response

Cells produce small protein molecules called cytokines, which have a specific effect on communication and interactions between cells. Other terms for cytokines include chemokine (cytokines with chemotactic characteristics), interleukins (cytokines generated by one leukocyte and acting on other leukocytes), monokines (cytokines produced by monocytes), and lymphokines (cytokines produced by lymphocytes) [141]. There are currently 18 cytokines known as interleukins (IL). Other cytokines, such as tumor necrosis factor (TNF), remain true to their original biological definition. Another aspect of several cytokines to consider is their function in infection and/or inflammation. While certain cytokines are known as pro-inflammatory cytokines because they significantly enhance inflammation, other group is characterized as anti-inflammatory cytokines since they counteract the effects of pro-inflammatory cytokines [142]. Cytokines can be categorized as pro-inflammatory or anti-inflammatory depending on their function [143]. Pro-inflammatory cytokines stimulate immune-competent cells and encourage inflammatory responses. Examples of these include IL-1, IL-6, IL-8, IL-12, TNF-, and interferons. On the other hand, anti-inflammatory cytokines, including TGF-*β*, IL-1 receptor antagonist (IL-1RA), IL-4, IL-6, IL-10, IL-11, and IL-13, suppress inflammation and inhibit immune cells [144].

## 5. Effect of Bisphenols on Alteration of Inflammation Markers

There is not much discussion in the scientific literature about the impact of bisphenols on changes in inflammatory markers. The authors illustrate the immunomodulatory impact of bisphenols using a variety of experimental animal and cell models. Cho et al., (2018) treated human endometrial stromal cell (ESC) cultures with 1000 pmol BPA [145]. The analyzed parameters were pro-inflammatory cytokines tumor necrosis factor (TNF), interleukin (IL)-6, and IL-1. After 4 h of treatment, there were no discernible changes in IL-1 levels; however, when the exposure period was extended to 48 h, the levels increased in a dose-dependent manner. BPA exposure dramatically increased the expression of inflammatory cytokine genes. Specific cytokine release was also elevated. In another study from 2013, Valentino et al., treated human adipocytes developed from 3T3-L1 cells with 1 nM of BPA for 24 h [146]. The results of this in vitro experimental study shown that IL-6 and IFN-γ levels were significantly higher in human and 3T3-L1 differentiated adipocytes. Other cytokines (IL-1b, IL-2, IL-4, IL-6, IL-10, GM-CSF, IFN-γ, KC/IL-8, MIP-1α, MIP-1β, RANTES, and TNF-α) did not vary substantially, except for MIP-1α, which was markedly increased in 3T3-L1 treated with BPA. One of the most important laboratory models for inflammatory process are human umbilical vein endothelial cells (HUVEC). Andersson and Brittebo (2012) cultivated HUVEC cells with 1 nM, 10 nM, and 1 μM BPA for 6 h. Endothelial cells incubated with 1 nM, 10 nM, and 1 μM BPA for six hours increased the mRNA expression of VEGF-A, VEGFR-2, eNOS, and Cx43 [147]. Cimmino et al. (2019) cultured human mature adipocytes and stromal vascular fraction cells with 0.1 nM BPA for 24 h [148]. The cells’ vitality was not affected by these concentrations. BPA boosted the production of IL-8, MCP1, and IL-6 in mature adipocytes as well as SVF cells. TNF and IL-1 were likewise elevated by BPA, but IL-10, which functions as an anti-inflammatory molecule in general, had the opposite trend. Interesting results were also obtained in studies by Xie et al. (2020) and Lee et al. (2020), where both author teams used RAW 264.7 macrophages as laboratory model [149,150]. One study used BPA treatment where expression of NLRP3 was reduced. The other study contained treatment with BPS, and after BPS treatment, the expression levels of inflammasome components NLRP3, ASC, cleaved caspase 1, and IL-1 rose considerably despite unaffected cell viability. Murine macrophage cells were cultivated with bisphenol S (10−3 M−10-10 M) for 30 min, 1 h, 6 h, and 24 h. Liu et al. (2014) also used macrophages (THP-1) and human peripheral blood mononuclear cells (PBMC) [151]. In this study, the results showed that BPA treatment at dosages ranging from 0 to 1 μM had no negative effects on THP1 macrophages over the 24 h incubation period when applied to THP1, a human monocytic leukemia cell line. The findings showed that BPA treatment of THP1 macrophages led to a little increase in IL-6 and significantly higher mRNA levels of the pro-inflammatory cytokine TNF-a. The observed rise in mRNA expression was correlated with the higher production of TNF-a and IL-6 proteins from THP1 macrophages following BPA treatment. On the other hand, BPA significantly decreased the expression of TGF-b, an anti-inflammatory cytokine. In vivo studies by Elswefy et al. (2016) and Yang et al. (2016) also showed increased pro-inflammatory cytokines (IL-1, IL-6) after BPA treatment using male Wistar albino rats (2–3 months old) and five-week-old C57BL/6J mice [152,153]. Most of the authors of the cited studies chose cytokines, mainly interleukins, as the major marker in their analyses. These studies prove the immunomodulatory effect of bisphenols, mainly by stimulating cytokine production.

## 6. Conclusions

Bisphenols are chemical compounds with some of the highest global production volumes. In the production of epoxy resins and polycarbonate polymers, many substances that are structurally identical to the majority bisphenol A are already used. Currently, it is reported that a total of 17 analogs of bisphenols have found wider industrial use, while the major bisphenols are BPA, BPS, BPF, BPB, and BPAF. Taking into account the endocrine-disrupting effects of these substances and their ubiquity, their influence on one of the basic immunological processes, inflammation, is a possible immunotoxic problem. According to the scientific literature, oxidative stress, ROS production, and epigenetic modifications are the primary mechanisms by which bisphenols affect the inflammatory response. The main three industrially used bisphenols (BPA, BPS, and BPF) changed the expression of both coding and non-coding RNAs during an analysis of the modifications of RNA profiles in human primary pre-adipocytes. After BPA, BPS, and BPF treatment, many authors have examined the differentially expressed RNAs and found that BPS and BPF caused comparable or more significant changes than BPA did. These chemicals’ endocrine-disrupting effects are also noteworthy in terms of general immunomodulation. In addition to oxidative stress, bisphenols disrupt hormone signaling, particularly the function of estrogen receptors (ERα and ERβ), which are expressed in endothelial cells. Bisphenols act as xenoestrogens binding to the ER, causing inappropriate activation or inhibition of downstream metabolic pathways. These substances activate pro-inflammatory pathways, such as nuclear factor kappa B (NF-κB), leading to increased expression of adhesion molecules (VCAM-1, ICAM-1) and cytokines (TNF-α, IL-6). Bisphenols have the ability to change how adhesion molecules are formed in endothelial cells, which can impact immune system cell infiltration. According to this perspective, the impact on the vascular and cellular responses—the two primary elements of inflammation—is significant. The summarized studies in the previous section show the immunomodulatory effect of bisphenols on various pro-inflammatory mechanisms. In the presented studies, the authors used animal laboratory models and cell cultures that are associated with inflammatory cascade. They mainly point to bisphenol’s effect of promoting and upregulating cytokine production. There are also hypotheses that bisphenols can alter cyclooxygenase activity, which is suggested to be the main method of inflammatory modification. Bisphenols activity may also alter prostaglandin production in an indirect way, because the entire inflammatory cascade represents a set of interconnected inflammatory mediators, the production of which influences each other. The cornerstone of these hypotheses is, in the vast majority of cases, associated with the stimulation of the production of pro-inflammatory cytokines and chemokines through epigenetic changes and stimulation of growth factors. In the scientific literature, this issue is still in the process of being resolved and needs strong support in specifying the specific mechanisms by which these substances influence the onset of inflammation at the molecular and metabolic level. The strong opinion about the immunomodulatory effect of bisphenols is related to the inflammatory process at the level of hypotheses. However, these hypotheses require concrete evidence about the metabolic pathways that are modulated by the influence of these substances.

Considering the complexity of the immune system and the immune response, it is necessary to focus not only on the well-described toxicity from the point of view of reproduction and the endocrine system, but also on the potential immunotoxicity of bisphenols, which is still not sufficiently clarified and represents a potential health risk.

Bisphenols can act on many inflammation markers and also can alter them, as many laboratory studies have shown. However, immunotoxic results from in vitro and in vivo studies are still only partial and need further addition in this scientific field.

The precise impact of bisphenols on the molecular mechanism of inflammatory markers remains in large part unclear. This fact is the main reason for the need for increased scientific awareness of the effects of bisphenols and their immunomodulatory effects and a new regulatory setting for BPA substituents.

## Data Availability

Data sharing is not applicable to this article as no new data were created or analyzed in this study.
